# Recent Advances in Traditional Chinese Medicine for Treatment of Podocyte Injury

**DOI:** 10.3389/fphar.2022.816025

**Published:** 2022-02-25

**Authors:** Tianwen Yao, Wenxiang Su, Shisheng Han, Yan Lu, Yanqiu Xu, Min Chen, Yi Wang

**Affiliations:** ^1^ Department of Nephrology, Yueyang Hospital of Integrated Traditional Chinese and Western Medicine, Shanghai University of Traditional Chinese Medicine, Shanghai, China; ^2^ Department of Nephrology, The People’s Hospital of Mengzi, Mengzi, China

**Keywords:** traditional Chinese medicine, podocyte injury, active ingredient, herbs, compound prescription, acupuncture and moxibustion

## Abstract

Podocyte is also called glomerular epithelial cell, which has been considered as the final gatekeeper of glomerular filtration barrier (GFB). As a major contributor to proteinuria, podocyte injury underlies a variety of glomerular diseases and becomes the challenge to patients and their families in general. At present, the therapeutic methods of podocyte injury mainly include angiotensin-converting enzyme inhibitors or angiotensin receptor blockers, steroid and immunosuppressive medications. Nevertheless, the higher cost and side effects seriously disturb patients with podocyte injury. Promisingly, traditional Chinese medicine (TCM) has received an increasing amount of attention from different countries in the treatment of podocyte injury by invigorating spleen and kidney, clearing heat and eliminating dampness, as well enriching qi and activating blood. Therefore, we searched articles published in peer-reviewed English-language journals through Google Scholar, PubMed, Web of Science, and Science Direct. The protective effects of active ingredients, herbs, compound prescriptions, acupuncture and moxibustion for treatment of podocyte injury were further summarized and analyzed. Meanwhile, we discussed feasible directions for future development, and analyzed existing deficiencies and shortcomings of TCM in the treatment of podocyte injury. In conclusion, this paper shows that TCM treatments can serve as promising auxiliary therapeutic methods for the treatment of podocyte injury.

## Introduction

Podocyte, also called glomerular epithelial cell, has been considered as the final gatekeeper of glomerular filtration barrier (GFB) (Yu et al., 2018). It is a kind of highly specialized cell that wraps around capillaries and neighbors the Bowman’s capsule. This special visceral epithelial cell contains multiple interdigitating foot processes separated by slit diaphragm, and plays a pivotal role in mediating cell signaling to maintain physiological functions (Inoue and Ishibe, 2015). Podocyte injury is characterized by dysfunction or structural damage of podocyte, which may be caused by different unknown factors, such as genetic disorders, immunological factors, inappropriate changes of renal hemodynamics, human immunodeficiency virus (HIV) infection, drugs with nephrotoxicity and heavy metal poisoning (Amiri, 2019). In fact, it has been frequently observed in clinical practice that podocyte injury will result in protein loss, which is the central event of various proteinuria. Besides, podocyte injury is recognized as a vital part in the pathogenesis of podocytopathies, and contributes to the occurrence and progression of chronic kidney disease (CKD) (Zuo et al., 2018). It is precisely because of podocyte injury that leads to the damage of GFB and gradually underlies the pathophysiology of various glomerulonephritis (Afsar et al., 2021). Furthermore, podocyte injury has already been regarded as a significant clinical prognostic index for glomerular diseases (Chen L. et al., 2017). So, preventing podocytes from being hurt is the central target to maintain renal function.

Although many breakthroughs in the fields of pharmacology and materials science have been produced for the treatment of podocyte injury, there is a lack of effective clinic-based treatments that can alleviate proteinuria and hypoalbuminemia caused by podocyte injury (Lu et al., 2019). Meanwhile, available treatments for clinical transformation still remain limited. Currently, angiotensin-converting enzyme inhibitors or angiotensin receptor blockers, steroid and immunosuppressive medications are key therapies to podocyte injury. These treatment modalities can partly reduce proteinuria, improve the survival rate and quality of life. However, part of them can cause high cost and result in a variety of side effects, including infection, bleeding and femoral head necrosis (Galati et al., 2021; Li T. et al., 2021). Furthermore, many patients also continue to progress to end-stage renal disease (ESRD) with those therapies, which can be a catastrophic event for the patient, family, and even society. Therefore, It is necessary to develop more potent, safe and economic therapies for podocyte injury.

Since Tu Youyou was awarded the 2015 Nobel Prize in Physiology and Medicine because of her discovery about a novel therapy against malaria called artemisinin, traditional Chinese medicine (TCM) has got increased attention (Kong and Tan, 2015). As a treasure house, the scientific nature of TCM derives from thousands of years of clinical application. It has spread from China throughout the whole Asia, to Africa, Europe and America (Gobe and Shen, 2015). TCM is based on the cumulative experience of previous practitioners, which is recorded detailedly in medical books by generations of doctors. The main principle of TCM is to regulate the balance of the yin and yang by focusing on the individualized care plan for each patient, which reflects the concept of personalized medicine. As is known, TCM can be divided into non-drug therapy and drug therapy. Among them, non-drug therapy mainly includes acupuncture and moxibustion, which requires only a pair of hands, some good quality acupuncture needles and moxa rather than oral medicines. Drug therapy has become more widely available throughout the world. In ancient times, one or two herbs were used in isolation to treat diseases. However, accumulated experience from clinical practice suggested that the combination of several herbs had greater efficacy than herbal agents in isolation, which was called prescriptions or formulas. With the development of advanced science and technology, more and more researchers try to study the underlying mechanisms of active ingredients that extracted from different herbs. Recently, the application of TCM to treat podocyte injury has received an increasing amount of attention from different countries. Although some studies indicate therapeutic benefits of TCM in models of podocyte injury, convincing evidence is limited. More importantly, the active ingredients and their underlying mechanisms remain unidentified. Fortunately, scientists from different countries in the field of TCM have made efforts to understand the cellular and molecular mechanisms of these Chinese herbs or formulas by using modern scientific techniques.

**FIGURE 1:**
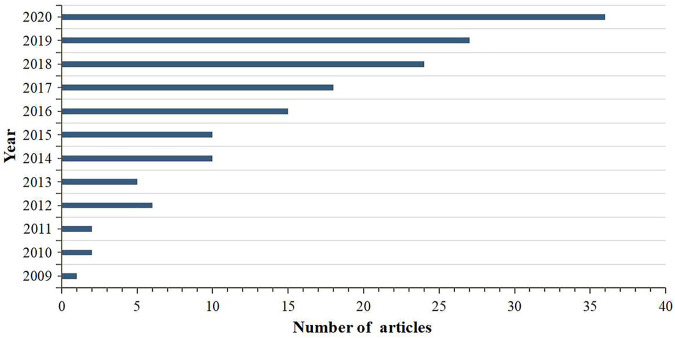
The number of articles on researches between TCM and podocyte injury published from 2009 to 2020.

We searched articles published in peer-reviewed English-language journals through Google Scholar, PubMed, Web of Science, and Science Direct using the following key words “traditional Chinese medicine,” “herbal medicine,” “traditional medicine,” “Chinese herbal compound,” “acupuncture,” “moxibustion,” and “podocyte” with a time frame between 2009 and 2020. Obviously, researches in the field of TCM and podocyte injury have gradually increased based on data from Figure 1. Besides, existing researches have demonstrated that active ingredients, Chinese herbal medicines, and TCM compounds exert varying degrees of therapeutic effects on podocyte injury (Yang et al., 2021a). This paper systematically reviews researches related to the use of TCM treatments for podocyte injury, summarizes feasible directions for future development, and analyzes existing deficiencies and shortcomings. We hope that our work will not only provide a broader discussion for the potential effects of TCM, but also help researchers in this field design and perform better studies in the future.

## Pathophysiology of Podocyte Injury

**FIGURE 2:**
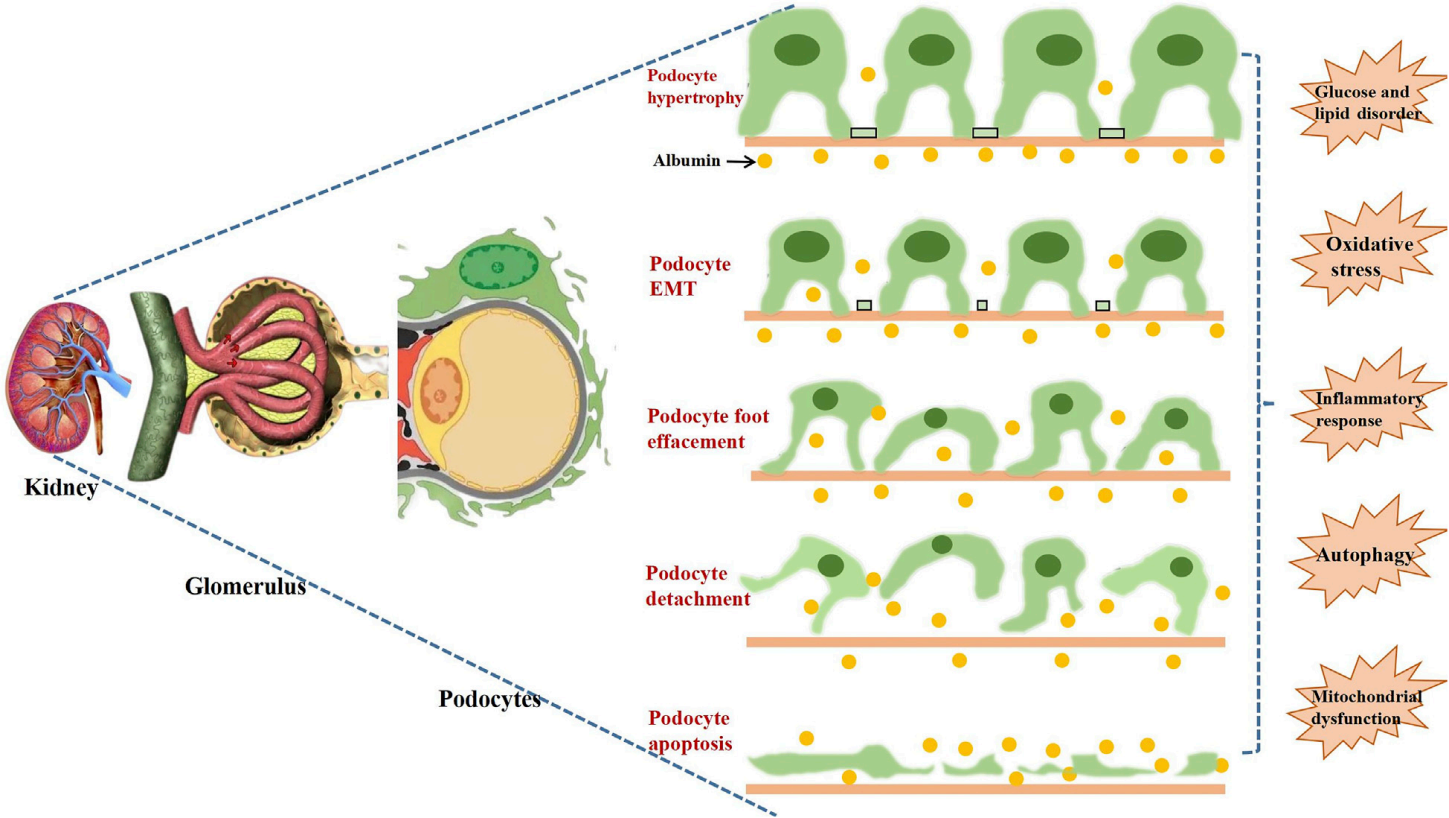
Different pathological processes of podocyte injury and its common factors.

Podocyte injury is the common pathological process in many glomerular diseases, such as minimal change disease (MCD), focal segmental glomerulosclerosis (FSGS), membranous nephropathy (MN), diabetic nephropathy (DN), lupus nephritis (LN), and other glomerulonephritis (Yoshimura and Nishinakamura, 2019). Podocyte lives in an environment of complex pathological stresses and stimuli, and adapts to maintain the integrity and stability of GFB (Nagata, 2016). However, excessive stresses will cause the maladaptation of podocyte and result in complex biological changes, mainly containing the loss of integrity and dysregulation of cellular metabolism. Then, injured podocyte will be associated with poor renal outcomes, such as increased proteinuria, glomerulosclerosis, and the deterioration of renal function. As shown in Figure 2, the following pathological processes represent the functional and morphological alterations of podocyte injury:

1) Podocyte hypertrophy is a crucial and early form of podocyte injury. In general, mature podocytes have to expand their sizes in order to compensate for glomerular dilation and cover the denuded regions of glomerular basement membrane (GBM) (Zhou et al., 2020).
2) The epithelial-mesenchymal transition (EMT) is usually considered as the central mechanism of podocyte injury. Podocytes lose their epithelial characteristics and gain the features of mesenchymal cells when the pathological process of EMT occurs, which induces disappeared cell contact and damaged cell polarity (Ying and Wu, 2017).
3) Podocyte foot effacement is the most significant morphological change of podocyte injury. Foot processes interdigitate with foot processes from adjacent podocytes and form a network of narrow and uniform gaps. The genetic or acquired impairment of podocytes may lead to foot effacement, which is a major cause of proteinuria (Reiser and Altintas, 2016).
4) Podocyte detachment is the hallmark of progressive glomerulosclerosis. The low expressions of podocyte marker proteins, such as synaptopodin, podocin and nephrin, will result in podocyte cytoskeleton disorder, damaged sufficient adhesion, and eventually lead to the detachment of podocytes from GBM (Gil et al., 2020).
5) Podocyte apoptosis plays an important role in reduction in density and number of glomerular. Under normal circumstances, apoptosis-promoting and anti-apoptosis signaling pathways coexist in the same condition to maintain the dynamic balance and guarantee the stable environment (Dai et al., 2017).

Although the pathogenesis of podocyte injury has not been fully understood, glucose and lipid metabolism disorder, oxidative stress (OS), inflammatory response, autophagy, and mitochondrial dysfunction have been widely accepted as the most common factors in the development of podocyte injury (Lin et al., 2021).

## Active Ingredients of TCM

**FIGURE 3:**
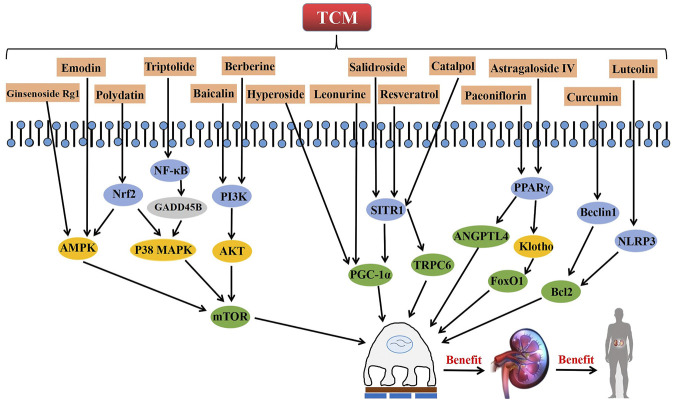
The role and related signaling pathways of active ingredients from TCM in the repair of podocyte injury.

Active ingredients associated with TCM are shown in Figure 3 and Table 1.

**TABLE 1:** Active ingredients of TCM potential therapeutic effects on podocyte injury.

Name	Source	Structure	Mechanism	Model	References
Astragaloside IV	*Astragalus mongholicus* Bunge (Fabaceae)	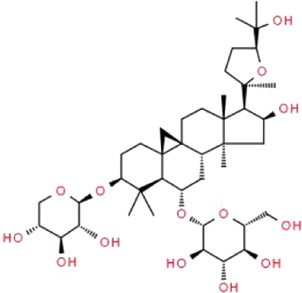	PPARγ-Klotho-FoxO1	db/db mice and HG-induced podocyte injury in cells; STZ-induced podocyte injury in rats and HG-induced podocyte injury in cells; KK-Ay mice and podocytes obtained from male C57BL/6J mice; PAN-induced podocyte injury in rats an cells	Gui et al. (2012), Guo et al. (2016), Yao et al. (2016), Wang X. et al. (2018), Zeng et al. (2020), Xing et al. (2021)
Triptolide	*Tripterygium wilfordii* Hook.f. (Celastraceae)	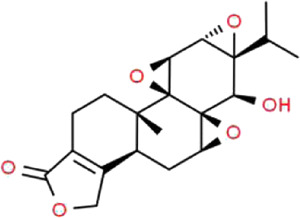	NF-κB/GADD45B/p38 MAPK	Zebrafish model of inducible podocyte-target injury; High-fat diet and STZ-induced podocyte injury in rats; ADR-induced podocyte injury in rats; TGF-β1-induced podocyte injury in cells; PM2.5-induced podocyte injury in cells	Ma et al. (2013), Jiang et al. (2015), Wang L. et al. (2018), Li C. et al. (2020), Wan et al. (2020), Wan et al. (2021)
Berberine	*Coptis chinensis* Franch. (Ranunculaceae)	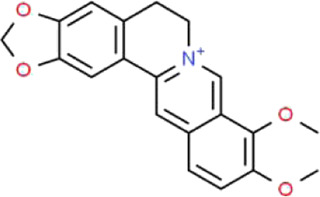	PI3K/AKT	STZ-induced podocyte injury in rats; Aldo-induced podocyte injury in rats and cells; Palmitic acid-induced podocyte in cells; HG-induced podocyte injury in cells	Wang et al. (2016), Ni et al. (2020), Li C.et al. (2020), Xiang et al. (2021)
Curcumin	*Curcuma longa* L. (Zingiberaceae)	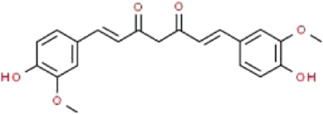	Beclin1/UVRAG/Bcl2	STZ-induced podocyte injury in rats and HG-induced podocyte injury in cells; Fructose-induced podocyte injury in rats; Ang II-induced podocyte injury in cells	Yu et al. (2020), Zhang et al. (2020), Ding et al. (2015)
Polydatin	*Reynoutria japonica* Houtt. (Polygonaceae)	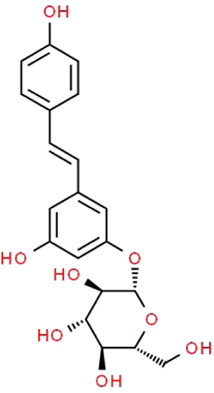	AMPK/p38 MAPK	Fructose-induced podocyte injury in rats; KK-Ay mice and HG-induced podocyte injury in cells	Zheng et al. (2017), Gu et al. (2021)
Emodin	*Rheum palmatum* L. (Polygonaceae)	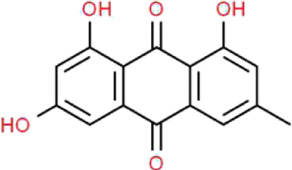	AMPK/mTOR	STZ-induced podocyte injury in rats and HG-induced podocyte injury in cells	Chen et al. (2015), Sohn et al. (2015), Liu H. et al. (2021)
Catalpol	*Rehmannia glutinosa* (Gaertn.) DC. (Orobanchaceae)	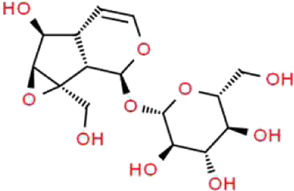	SIRT1/MRP2/TRPC6	ADR-induced podocyte injury in rats and cells; STZ-induced podocyte injury in rats and HG-induced podocyte injury in cells	Chen et al. (2019a), Chen et al. (2019b), Zhang J. et al. (2019)
Silybin	*Silybum marianum* (L.) Gaertn. (Asteraceae)	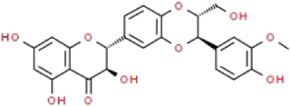	-	OVE26 mice and HG-induced podocyte injury in cells	Khazim et al. (2013)
Luteolin	*Lonicera japonica* Thunb. (Caprifoliaceae)	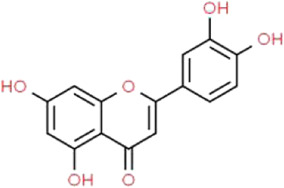	NLRP3 inflammasome	HG-induced podocyte injury in cells	Yu et al. (2019)
Hyperoside	*Eleutherococcus senticosus* (Rupr. & Maxim.) Maxim. (Araliaceae) and *Forsythia suspensa* (Thunb.) Vahl (Oleaceae)	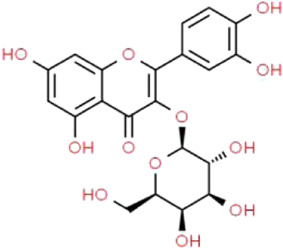	PGC-1α	ADR-induced podocyte injury in rats and cells; STZ-induced podocyte injury in rats and HG-induced podocyte injury in cells; CoCl_2_-induced podocyte injury in cells	Zhang et al. (2016), An et al. (2017), Chen L. et al. (2017), Wu et al. (2019)
Leonurine	*Leonurus cardiaca* L. (Lamiaceae)	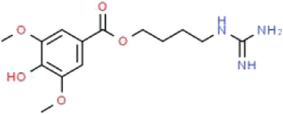	PGC-1α-mitochondria	ADR-induced podocyte injury in rats and cells	Liu X. et al. (2018)
Salidroside	*Rhodiola rosea* L. (Crassulaceae)	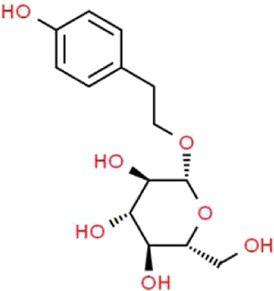	SIRT1/PGC-1α	STZ-induced podocyte injury in rats; HG-induced podocyte injury in cells; ADR-induced podocyte injury in rats	Lu H. et al. (2017), Huang et al. (2019), Xue et al. (2019)
Paeoniflorin	*Paeonia lactiflora* Pall. (Paeoniaceae)	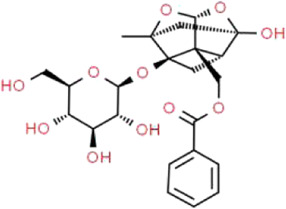	PPARγ/ANGPTL4	ADR-induced podocyte injury in rats and cells	Lu R. et al. (2017)
Resveratrol	*Reynoutria multiflora* (Thunb.) Moldenke (Polygonaceae)	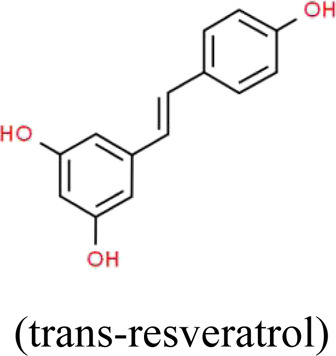 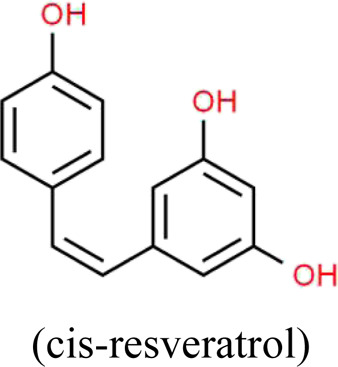	SIRT1/PGC-1α	STZ-induced podocyte injury in rats and HG-induced podocyte injury in cells; High-fat diet and ovariectomy- induced podocyte injury in rats; Non obesity diabetes rats	Xian et al. (2019), Zhang T. et al. (2019), Li B. et al. (2020)
Ginsenoside Rg1	*Panax ginseng* C.A.Mey. (Araliaceae)	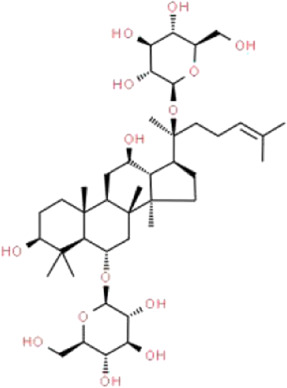	AMPK/mTOR/PI3K	Ang II-induced podocyte injury; STZ-induced podocyte injury in rats and HG-induced podocyte injury in cells	Mao et al. (2016), Shi et al. (2020), Wang et al. (2020)
Baicalin	*Scutellaria baicalensis* Georgi (Lamiaceae)	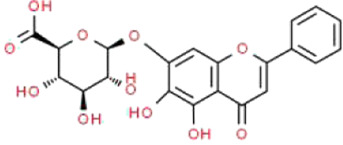	PI3K/AKT/mTOR	High-fat diet and STZ-induced podocyte injury in rats; HG-induced podocyte in cells; ADR-induced podocyte injury in rats and TGF-β1-induced podocyte in cells	Li J. et al. (2020), Dou et al. (2020), Qu et al. (2021)

### Astragaloside IV

Astragaloside IV (AS-IV), a small active substance extracted from Chinese herbal medicine *Astragalus mongholicus* Bunge (Fabaceae) (Shi et al., 2021), presents diverse pharmacological properties of anti-oxidation, anti-inflammation, anti-cancer, anti-apoptosis and immune-regulation (Zhu et al., 2021). It has been demonstrated that AS-IV possesses potent protective effects against diabetes, cancer, kidney diseases, cardiovascular diseases, pulmonary diseases, liver fibrosis, cognitive diseases, inflammatory and autoimmune diseases (Li L. et al., 2017). Gui et al. reported that AS-IV prevents podocyte apoptosis, reduces albuminuria and OS in streptozotocin (STZ)-induced rats and high glucose (HG)-induced cells through decreasing the expression of Bax and inhibiting the activation of caspase-3 (Gui et al., 2012). Yao et al. found that AS-IV protects HG-induced podocyte apoptosis *via* down-regulating TRPC6 expression and suppressing intracellular Ca^2+^ in podocytes (Yao et al., 2016). In addition, Guo et al. demonstrated that AS-IV restores intracellular Ca^2+^ homeostasis and attenuates podocyte apoptosis in a dose-dependent manner with a concomitant abrogation of endoplasmic reticulum stress (ERS) (Guo et al., 2016). Wang et al. showed that AS-IV inhibits the over-expression of miR-21, ameliorates renal fibrosis and decreases the dedifferentiation of podocytes (Wang X. et al., 2018). Xing et al. concluded that AS-IV protects against podocyte apoptosis in DN by inhibiting OS, which is closely related to the activation of PPARγ-Klotho-FoxO1 signaling pathway (Xing et al., 2021). AS-IV also reportedly improves the expressions of Wnt5a, protein tyrosine kinase 7, Rho-associated coiled-coil-containing protein kinase 1, and Ras-related C3 botulinum toxin substrate 1, thereby restoring the distribution of F-actin and synaptopodin in PAN-induced podocyte injury (Zeng et al., 2020).

Of the various active ingredients extracted from Chinese herbal medicines, AS-IV has considerable potential for the treatment of podocyte injury. It has participated in the complex pathophysiological processes of podocyte injury and regulates multiple signal pathways in a variety of kidney diseases. Therefore, high quality clinical trials about AS-IV are important in the near future. Because the correct dosage and administration of AS-IV have not been standardized, a long-term reasonable approach to the safe treatment is also needed. Furthermore, its side effects have not yet been determined, which may influence the wide application of AS-IV.

### Triptolide

Triptolide (TP) is an important bioactive diterpenoid extracted from the root of traditional Chinese herb *Tripterygium wilfordii* Hook.f. (Celastraceae) (Huo et al., 2021), which is also known as “thunder god vine.” It has a long history of use in China primarily in the treatment of inflammatory and autoimmune diseases (Luo et al., 2019). Furthermore, TP also exhibits multiple pharmacological activities, including anticancer, anti-proteinuric, anti-fibrosis and neuroprotective properties (He D. et al., 2021). Ma et al. found that TP can attenuate albuminuria and improve podocyte injury in the rat model of DN by inhibiting macrophage infiltration, while significantly decreasing the secretion of inflammatory cytokines in the kidneys (Ma et al., 2013). Jiang et al. suggested that the therapeutic effects of TP on ADR-induced podocyte injury are achieved through regulating the expressions of miRNA-344b-3p and miRNA-30b-3p (Jiang et al., 2015). Recently, it is reported that the therapeutic value of TP in TGF-β1-induced podocyte injury might involve the progress of podocyte EMT process and TET2-mediated DNA demethylation (Wan et al., 2021). Wan et al. demonstrated that the administration of TP ameliorates PM2.5-induced podocyte injury, which is associated with suppressing the expressions of Bax, NF-κB/p65 and p-IκBα (Wan et al., 2020). Wang et al. proposed that TP protects podocytes from apoptosis *via* the inhibition of NF-κB/GADD45B/p38 MAPK pathway (Wang L. et al., 2018).

Although TP exhibits efficacy as a therapeutic agent for podocyte injury, research results regarding TP still remain limited. For example, the roles of TP in the different stages of podocyte injury and the specific molecular mechanisms underlying these processes both require further analyses. Besides, the toxic effects of TP on the heart, liver, and kidneys limits its clinical application. Therefore, clinical use of TP for podocyte injury will require a long and in-depth research process that includes quantitative studies to assess the efficacy and security of TP.

### Berberine

Berberine (BBR) is a famous alkaloid mainly extracted from *Coptis chinensis* Franch. (Ranunculaceae), which is also known as an ancient herbal medicine in treating diarrhea called Huang lian (Wong et al., 2021). As a medication and functional food additive, it exerts therapeutic effects in TCM for the treatment of various diseases, including diabetes, infection, dementia, arrhythmia and cancer (Zhang Y. et al., 2021). It is reported that BBR has pharmacological activities of anti-inflammation, antioxidant, anticonvulsant, antidepressant, anti-alzheimer, anti-cancer, anti-fibrosis and anti-osteoporosis, which make it increasingly become a hotspot of research (Zhou M. et al., 2021). Wang et al. showed that BBR promotes the repair of RAAS activation-induced podocyte injury by suppressing OS and ERS (Wang et al., 2016). Likewise, Xiang et al. reported that ERS and increased production of reactive oxygen species (ROS) are the key points of podocyte injury, and BBR protects against palmitic acid-induced podocyte apoptosis by suppressing ROS-dependent ERS (Xiang et al., 2021). Additionally, Ni et al. found that BBR provides important positive effects for the treatment of podocyte injury by significantly improving the abnormal expressions of PI3K, AKT and phosphorylated AKT in STZ-induced rats (Ni et al., 2020). They also proved that the possible mechanism of BBR might be associated with the PI3K-AKT pathway. Li et al. demonstrated that BBR activates podocyte autophagy through inhibiting mTOR and the phosphorylation of P70S6k and 4EBP1 in podocytes under the HG condition (Li C. et al., 2020).

Currently, BBR is extensively used in TCM with various beneficial properties and gradually acts as a potential candidate in the treatment of podocyte injury. However, its effects and the underlying mechanisms are not fully understood. These problems largely limit the application of BBR. Therefore, elucidating the molecular mechanism of BBR in the treatment of podocyte injury is an important research direction in the future.

### Curcumin

Curcumin (CUR), a bright yellow chemical, is the main component of turmeric isolated from the rhizomes of *Curcuma longa* L. (Zingiberaceae) (Yarmohammadi et al., 2021)*.* It has been used for medicinal purposes for thousands of years with its biological and pharmacological properties, including anti-inflammatory, anti-oxidation, immunomodulatory, antitumor, antibacterial, antiapoptotic (Li Y. et al., 2021). Additionally, it is also frequently applied as a popular fragrance, coloring, and additive in Asia. Therefore, the immense promise of CUR has already been established in the treatment and clinical management of various chronic diseases, such as cancer, metabolic diseases, cardiovascular diseases, neurological diseases and inflammatory diseases (Kunnumakkara et al., 2019). Ding et al. reported that CUR reduces proteinuria in fructose-induced rats and protects against podocyte injury by improving insulin signaling and activating miR-206 expression (Ding et al., 2015). Yu et al. showed that CUR attenuates Ang II-induced podocyte apoptosis through inhibiting ERS. In addition, They found CUR reduces the levels of nephrin, podocin, synaptopodin and F-actin in a dose-dependent manner (Yu et al., 2020). Zhang et al. suggested that CUR increases podocyte marker proteins in STZ-induced rats, improves the viabilities and inhibits the apoptosis of podocytes exposed to HG *via* the regulation of Beclin1/UVRAG/Bcl2 pathway (Zhang et al., 2020).

Although the protective effects of CUR on podocytes exhibit an obvious dose-response correlation, this relationship has not been completely confirmed, and the most appropriate concentration remains unclear. Furthermore, it needs to be considered that under the same dose regimen, whether different patients can achieve satisfactory therapeutic efficacy. So, these scientific hypotheses or issues must be supported by strong experimental evidences.

### Polydatin

Polydatin (PD), a natural compound extracted from the roots of traditional Chinese herbal medicine *Reynoutria japonica* Houtt. (Polygonaceae), can also be found in daily foods such as grapes and red wine (Liu T. et al., 2021). Various studies have shown that PD has pharmacological effects on ischemia-reperfusion injuries of kidneys, heart, cerebra, lungs and other organs (Sun and Wang, 2020). Furthermore, it is also known to have anti-inflammatory, antioxidative, anti-apoptotic, hepatoprotective and immunoregulatory effects (Ebrahim and Elsherbini, 2021). Zheng et al. demonstrated that PD stabilizes mitochondrial morphology, and attenuates mitochondrial malfunction in both KK-Ay mice and HG-induced cells through suppressing Drp1 expression (Zheng et al., 2017). Gu et al. showed that PD has protective effects on fructose-induced podocyte injury through suppressing autophagy and alleviating OS. Furthermore, those positive effects are achieved by the enhancement of Nrf2-dependent antioxidant capacity and the inhibition of AMPK/p38 MAPK signaling (Gu et al., 2021). Although there is no high-quality direct data evidences that PD can improve diverse podocyte injuries, it is still reasonable to expect that PD plays a potential therapeutic role in the progress of podocyte repair.

### Emodin

As a natural anthraquinone derivative, emodin (EMO) is one of the main active ingredients in the root and rhizome of *Rheum palmatum* L. (Polygonaceae), which is also called “General” in TCM. It is recognized as a protein tyrosine kinase inhibitor and attacks against various tumour cells, including breast, liver, lung and ovarian cancer cells (Tuli et al., 2021). Additionally, EMO is capable of inducing fibroblast apoptosis and improving the lipid accumulation for its anti-inflammatory, antioxidative, hepatoprotective, neuroprotective, antimicrobial and antifibrotic activities (Semwal et al., 2021). Chen et al. found that EMO significantly ameliorates albuminuria, inhibits desmin expression, preserves nephrin expression in STZ-induced rats, as well as improving HG-induced EMT and subsequent podocyte dysfunction (Chen et al., 2015). Sohn et al. demonstrated that EMO reduces proteinuria, prevents podocyte loss, and exerts an MGO scavenging effect in STZ-induced rats through inhibiting MGO-derived advanced glycation end-product formation (Sohn et al., 2015). Liu et al. reported that EMO exerts renoprotective effects in STZ-induced rats including suppressing cell apoptosis and enhancing autophagy of podocytes, which is known to involve the AMPK/mTOR signaling pathway in the kidney (Liu H. et al., 2021). Although current researches indicate that EMO performs various biological activities and promotes the repair of podocyte injury in DN, meaningful clinical studies are still expected to be completed. Thus, the therapeutic effects of EMO warrant further investigation considering its possible side effects for the patient.

### Catalpol

Catalpol (CAT) is a natural iridoid glycoside and acts as a major effective constituent extracted from *Rehmannia glutinosa* (Gaertn.) DC. (Orobanchaceae) (Kim et al., 2020). Numerous studies have confirmed that CAT has a wide range of biological activities, including antioxidative, anti-inflammatory, anti-apoptosis, and anti-fibrosis properties (Bhattamisra et al., 2020; He L. et al., 2021). It is generally accepted that CAT exerts therapeutic effects on psoriasis, cerebrovascular diseases, neuromuscular diseases and fatty liver disease (Tian et al., 2020). Chen et al. found that CAT significantly increases cell viability, SOD activity, and decreases LDH release, ROS generation, levels of MDA and inflammatory cytokines in HG-induced podocyte injury (Chen et al., 2019a). Furthermore, they demonstrated that CAT improves the expressions of nephrin and synaptopodin, rescues injured cytoskeleton, increases migration ratio in podocytes induced by HG, and the protective effect of is closely related to the improvement of podocyte autophagy and stabilization of cytoskeleton structure (Chen et al., 2019b). Zhang et al. proved that CAT exhibits strong protective effects against ADR-induced nephropathy both *in vitro* and *in vivo* by enhancing the expressions of SIRT1 and MRP2, as well as inhibiting the expression of TRPC6 (Zhang J. et al., 2019). Although the most appropriate dose of administration for CAT still remains unknown and the relevant molecular mechanisms have not yet been fully elucidated, it is inspiring that those current evidences will encourage new discoveries in this area.

### Silybin

Silybin, also known as silibinin, is the most abundant and major active ingredient of silymarin. It is extracted from a medicinal plant called *Silybum marianum* (L.) Gaertn. (Asteraceae) (Shin et al., 2015)*.* Bijak demonstrated that the molecular structure of silybin consists two main units, including taxifolin and phenyllpropanoid, and they are tightly linked together into one structure by a ring (Bijak, 2017). Silybin has been regarded as an important substance with hepatoprotective property in the therapies of diverse liver diseases (Zhang R. et al., 2021). Besides, silybin has been shown to provide other biological functions, such as anticancer, antioxidative, anti-fibrotic and anti-inflammatory activities. Khazim et al. showed that antioxidant silybin can reduce proteinuria in the OVE26 mice and protect against HG-induced podocyte injury (Khazim et al., 2013). Furthermore, they reported that the therapeutic effects of silybin on podocyte injury are related to the antioxidative and anti-inflammatory signaling pathways, which may not be limited to the inhibition of NADPH oxidase activity. Although high quality clinical trials are not conducted, and the relevant underlying mechanisms have not yet been fully elucidated, current studies support the concept that silybin may be a novel therapeutic intervention for the treatment of DN.

### Luteolin

As one of the most bioactive flavonoids, luteolin (LUT) is a kind of natural ingredient extracted from a variety of medicinal plants, fruits and vegetables, such as *Lonicera japonica* Thunb. (Caprifoliaceae) (Tesio and Robledo, 2021). Recently, the increasing reduction in the application of synthetic antioxidants makes the study of LUT a very active field. LUT has great pharmacological effects such as anti-diabetic, hepatoprotective, anti-inflammation, antioxidative, anti-tumor, and neuroprotective activities (Manzoor et al., 2019; Ashrafizadeh et al., 2020). Yu et al. found that LUT alleviates podocyte apoptosis from HG-induced injury by inhibiting the activation of NLRP3 inflammasome (Yu et al., 2019). Unfortunately, there is a lack of further information and studies regarding the effects of LUT on podocyte injury. However, NLRP3 inflammasome has been reported to play a crucial role in DN, and it is a commonly assessed signaling target in studies of podocyte injury (Wen et al., 2021). Thus. the value of the study regarding the importance of LUT in the treatment of podocyte injury is worth affirming.

### Hyperoside

Hyperoside (HPS) is an active flavonoid glycoside extracted from many Chinese herbal medicines, including *Eleutherococcus senticosus* (Rupr. & Maxim.) Maxim. (Araliaceae) and *Forsythia suspensa* (Thunb.) Vahl (Oleaceae). It has protective effects on several diseases in clinical practice, such as myocardial infarction, neuroinflammation, cognitive impairment and fatty liver disease (Chen X. et al., 2021; Sun et al., 2021; Yang et al., 2021b). Zhang et al. successfully provided experimental evidence about the therapeutic effects of HPS on proteinuria, slit diaphragm protein nephrin and podocin in STZ-induced DN at the early stage (Zhang et al., 2016). An et al. found that HPS also has a prophylactic effect on the progression of proteinuria and GBM damage in DN through inhibiting podocyte heparanase expression (An et al., 2017). Chen et al. reported that HPS blocks ADR-induced mitochondrial dysfunction and podocyte injury by increasing the expression of PGC-1α and inhibiting mitochondrial fission both *in vivo and in vitro*, which supports protective effects of HPS on various forms of glomerular diseases (Chen Z. et al., 2017). Furthermore, Wu et al. demonstrated that HPS inhibits CoCl_2_-induced mitochondrial fission, attenuates OS and apoptosis by regulating OMA1-OPA1 axis (Wu et al., 2019). Thus, HPS may be a promising therapeutic strategy in the treatment of podocyte injury.

However, a pharmacokinetic study indicated that unbound HPS exists in rat blood circulation and kidney after oral administration, which suggests that the effect of HPS may depend on its original types, rather than its metabolites (Xue et al., 2011). So, to clarify the protective effects and detail molecular mechanisms of HPS on podocyte injury, we need to further investigate the best original type of HPS in future work.

### Leonurine

Leonurine is the main active ingredient from *Leonurus cardiaca* L. (Lamiaceae), which has been proved to have anti-inflammatory and antioxidative properties (Xu et al., 2014). It shows therapeutic effects in several diseases, including AKI, renal fibrosis, cardiovascular diseases, diabetes and depression (Cheng et al., 2015). Furthermore, Liu et al. demonstrated that leonurine treatment significantly prevents early kidney damage, reduces macrophage infiltration and proteinuria (Liu X. et al., 2018). Meanwhile, the application of leonurine suppresses ADR-induced podocyte injury and inhibits OS *via* the regulation of PGC-1α-mitochondria axis. Although there is a relative lack of researches regarding the effects and mechanisms of leonurine, it might be a promising and important therapeutic drug for the prevention of podocyte injury and other kidney diseases.

### Salidroside

Salidroside (SAL) is the main active ingredient of *Rhodiola rosea* L. (Crassulaceae), which is first recorded in the Four Pharmacopoeia of Tibetan medicine, and gradually becomes one of the most commonly used Chinese herbal medicines in China. As a well-known antioxidant, SAL is capable of exerting diverse pharmacological activities, including antioxidative, anti-inflammatory, anti-cancer, anti-aging and anti-fibrosis properties (Huang et al., 2021; Song et al., 2021). Lu et al. showed that SAL reduces HG-induced OS, decreases podocyte apoptosis, and improves podocyte viability by up-regulating the expression of HO-1 (Lu H. et al., 2017). Huang et al. concluded that SAL ameliorates proteinuria, improves expressions of nephrin and podocin, reduces kidney fibrosis and podocyte injury in ADR-induced nephropathy, which may rely on the inhibition of β-catenin (Huang et al., 2019). Xue et al. found that SAL attenuates foot process effacement and improves mitochondrial biogenesis against podocyte injury in diabetic mice *via* SIRT1/PGC-1α axis (Xue et al., 2019).

Although those studies suggest that SAL plays a potential role in protecting podocytes through anti-oxidative and anti-hyperglycemia activities, the therapeutic effects in clinical remain uncertain. Thus, high quality clinical researches need to be conducted to determine whether SAL is effective in the treatment of podocyte injury. Besides, the underlying mechanisms of SAL in suppressing podocyte injury are complex and further studies both *in vivo* and *in vitro* should be completed. For example, cell experiments about podocyte injury are needed to verify the present findings.

### Paeoniflorin

Paeoniflorin (PF) is an effective water-soluble monoterpene glycoside extracted from the dried root of *Paeonia lactiflora* Pall. (Paeoniaceae). Except anti-depressive property, PF also contributes to the promotion of neuroprotection, down-regulation of nitric oxide level, inhibition of inflammatory reaction, fibrosis, and apoptosis (Jiao et al., 2021). So, it is widely used in various Chinese herbal formulas for treating depression, cardiovascular diseases, neurological ailments and kidney diseases (Wang et al., 2021). Meng et al. found that PF, as one of the effective bioactive compositions for the treatment of chronic renal failure, ameliorates microinflammation status, retards OS and finally alleviates podocyte injury (Meng et al., 2013). Lu et al. showed that PF obviously increases the expression of synaptopodin and decreases the expression of desmin, demonstrating a role in protecting against ADR-induced nephropathy both *in vivo* and *in vitro* (Lu R. et al., 2017). Meanwhile, this study provides the evidence that PF may exert therapeutic effects in podocyte injury and renal dysfunction by activating PPARγ and subsequently inhibiting ANGPTL4.

It is believed that PF, as a significant source of natural treatment strategies, has potential values in the treatment of various kidney diseases associated with podocyte injury. However, clinical researches in this area still remain very limited, and extensive in-depth experimental studies are needed to accelerate the development of PF in this field.

### Resveratrol

Resveratrol (RES) is a natural polyphenolic ingredient originally extracted from the roots of *Reynoutria multiflora* (Thunb.) Moldenke (Polygonaceae) and other herbs (Agah et al., 2021), which possesses potential antioxidative, anticancer and anti-inflammatory effects (Kohandel et al., 2021). It can also be found in red wine, peanuts, and grape skins. A growing body of evidences indicate that RES plays an important role in the treatment and prevention of different diseases, including cancer, cardiovascular diseases, obesity and age-related diseases (Fraiz et al., 2021; Ratajczak and Borska, 2021). Li et al. found that RES significantly attenuates obesity-associated early podocyte injury through improving lipid metabolism and insulin sensitivity, as well as inhibiting inflammatory responses in rats induced by an ovariectomy with a HFD (Li B. et al., 2020). Xian et al. reported that the combination of hUCMSCs and RES can reduce BG, BUN, SCr, the expressions of inflammatory factors MCP-1, NF-кB, and increase the number of podocytes and the expression of the podocyte-related proteins in NOD mice, which indicates a novel therapeutic method for the treatment of podocyte injury (Xian et al., 2019). Similarly, Zhang et al. showed that RES ameliorates podocyte injury through inhibiting mitochondrial OS and apoptosis both *in vivo* and *in vitro*, which is closely associated with SIRT1/PGC-1α pathway (Zhang T. et al., 2019).

The antioxidative efficacy of RES holds promise for its potential use in the treatment of a variety of diseases associated with OS, including podocyte injury. However, clinical and experimental researches in this area still remain lower influence, particularly in early prevention and treatment of podocyte injury. Further studies no doubt need to be done to evaluate the proper application of RES.

### Ginsenoside Rg1

Ginsenoside Rg1 (Rg1), a glycosylated triterpenoid saponin, is the main bioactive constituent extracted from *Panax ginseng* C.A.Mey. (Araliaceae) (Xu et al., 2021). As is known, *Panax ginseng* C.A.Mey. (Araliaceae) is a valuable Chinese herbal medicine used for Qi deficiency, which has been widely cultivated in China and other countries. Rg1 has been reported to affect various human organ systems including the immune, cardiovascular, kidney and nervous systems with its pharmacological properties of antioxidative, anti-inflammatory, antidepressant, and anticancer effects (Xie et al., 2018a). Shi et al. showed that Rg1 significantly alleviates renal fibrosis and podocyte EMT both in STZ-induced rats and podocytes exposed to HG by restoring autophagic activity (Shi et al., 2020). Wang et al. suggested that the therapeutic effects of Rg1 are related to the inhibition of pyroptosis through suppressing hyperlipidemia-induced NLRP3 inflammasome in podocytes (Wang et al., 2020). Mao et al. found that Rg1 significantly inhibits the formation of autophagosomes and attenuates the expression of autophagy-related proteins in Ang II-induced podocytes, which is achieved through the regulation of AMPK/mTOR pathway (Mao et al., 2016).

Thus far, Rg1 has been shown to exert protective effects in different models both *in vivo* and *in vitro*. However, there is no obvious correlation between the protective effects of Rg1 and the models of podocyte injury. Meanwhile, existing experimental results are inconclusive and further researches with persuasive results are needed to support the clinical use of Rg1. Accordingly, Rg1 is expected to become an important supplementary drug for the treatment of podocyte injury.

### Baicalin

Baicalin (BA) is a major flavonoid extracted from the root of *Scutellaria baicalensis* Georgi (Lamiaceae), which is called Huang qin in TCM (Chang et al., 2021). Recently, numerous studies have shown that BA has salutary effects for anti-inflammatory, antibacterial, antioxidative, anti-apoptotic, immunomodulatory, and anti-atherosclerotic properties (Li Y. et al., 2020). With those properties, BA has been demonstrated to exert beneficial therapeutic applications in pneumonia, diabetes, cardiovascular diseases, inflammatory bowel diseases, asthma, neurological diseases and kidney diseases (Dinda et al., 2017). Li et al. reported that treatment with BA can significantly promote the viability of podocytes, decrease HG-induced podocyte apoptosis, and finally serve a protective role in a dose-dependent manner (Li J. et al., 2020). Dou et al. investigated the effects of BA on ADR-induced rats and TGF-β1-induced podocytes, and they found that BA reduces proteinuria, relieves glomerulus structural disruption and dysfunction by inhibiting the expressions of podocyte EMT markers and cell migration (Dou et al., 2020). Qu et al. proposed that BA can slow down the damage of podocytes caused by hyperglycemia, and the underlying mechanism is closely related to the suppression of PI3K/AKT/mTOR signaling pathway (Qu et al., 2021).

Although BA is a commonly used clinical drug that has benefited many patients with respiratory system diseases, its use is limited in terms of podocyte injury. There is a relative lack of research results regarding the specific mechanisms underlying these processes. In addition, the protective effects of BA on podocytes are still doubtful based on the current literature. Therefore, more in-depth basic and clinical studies are required to solve above problems. It is firmly believed that BA can be used as an effective supplementary therapy for podocyte injury in the future.

## Herbs of TCM

Chinese herbal medicines associated with podocyte injury are shown in Table 2. The pictures of relevant herbs are obtained from Plants of the World Online database (http://www.plantsoftheworldonline.org/).

**TABLE 2:** Herbs of TCM potential therapeutic effects on podocyte injury.

Name	Picture	Effect in TCM	Main active ingredients	Mechanism	Model	References
*Astragalus mongholicus* Bunge (Fabaceae)	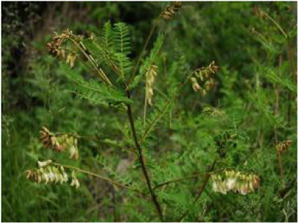	Tonifying qi and strengthening exterior	Atragaloside I–IV, polysaccharides, amino acids and trace elements	-	STZ-induced podocyte injury in rats	Zhai et al. (2019)
*Cordyceps sinensis* (BerK.) Sacc. Mycelia (Clavicipitaceae)	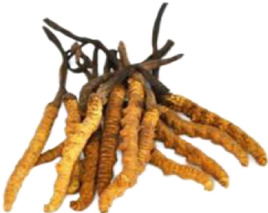	Reinforcing kidney and nourishing lung	Cordycepin, arachidonic acid, cerevisterol, beta-sitosterol, linoleyl acetate, cholesteryl palmitate	p38/JNK	C5b-9-induced podocyte injury in cells; STZ-induced podocyte injury in rats; High-fat diet with STZ-induced podocyte injury in rats and HG-induced podocyte injury in cells	Hao et al. (2014), Hong et al. (2015), Wang C. et al. (2018)
*Salvia przewalskii* Maxim. (Lamiaceae)	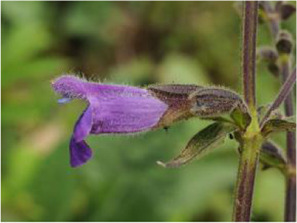	invigorating blood circulation and eliminating stasis	tanshinone, isotanshinone, danshenxinkun, danshenol and tanshinlactone	TLR4	PAN-induced podocyte injury in rats and cells	Dai et al. (2015), Liu et al. (2016), Ren et al. (2018)
*Schisandra chinensis* (Turcz.) Baill. (Schisandraceae)	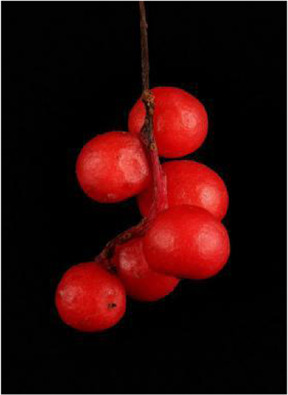	Tonifying qi and strengthening yin	Schisandrin B, schisandrol B, anwuligan, schisanhenol and deoxyschizandrin	-	STZ-induced podocyte injury in rats and TGF-β1-induced podocyte injury in cells	Zhang et al. (2012)
*Kopsia arborea* Blume (Apocynaceae)	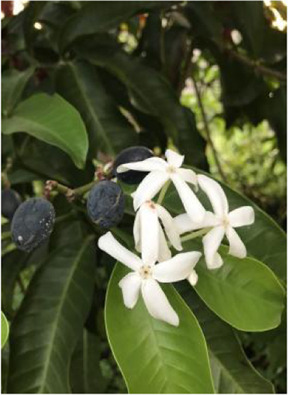	Clearing heat and removing toxicity	Kopsioffines A-C, kopsiofficines H-L, erythratinone, erythratine, erysotine, cristanine E, kopsinidines C-E, 12-methoxychanofruticosinic acid, chanofruticosinic acid, methyl chanofruticosinate, erthrodiol and β-amyrin	-	HG-induced podocyte injury in cells	Wang et al. (2019)
*Atractylodes lancea* (Thunb.) DC. (Asteraceae)	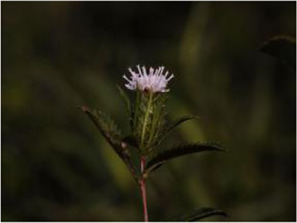	Invigorating spleen for eliminating dampness	Atractylon, atractyloside A, tractylenolides I, II, III and 8-epiasterolid	TRPC6/p-CaMK4	Fructose- induced podocyte injury in rats	Chen L. et al. (2021)
*Panax notoginseng* (Burkill) F.H.Chen (Araliaceae)	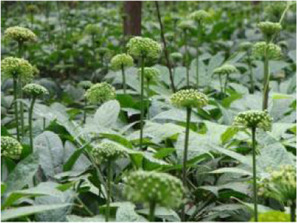	Promoting blood circulation, stopping bleeding, relieving swelling and pain	Notoginsenosides, triterpenoid saponins and dencichine	Wnt/β-catenin	STZ-induced podocyte injury in rats	Xie T. Z. et al. (2020)
*Abelmoschus manihot* (L.) Medik. (Malvaceae)	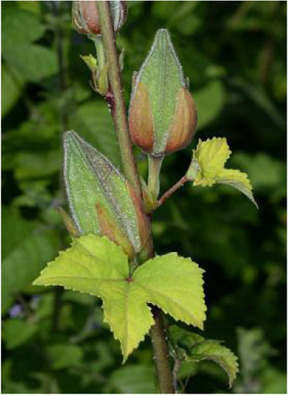	Clearing heat and promoting diuresis	Flavonoids, amino acids, polysaccharides, steroids, organic acids and volatile oils	p38MAPK; PTEN/PI3K/Akt	ADR-induced podocyte injury in rats; HG-induced podocyte injury in cells; STZ-induced podocyte injury in rats	Tu et al. (2013), Kim et al. (2018), Liu B. H. et al. (2021)

### *Astragalus mongholicus* Bunge (Fabaceae)

The medicinal herb *Astragalus mongholicus* Bunge (Fabaceae) (AS)*,* commonly known as Huang qi in TCM, can be used as medicine and food. For thousands of years, AS has been applied frequently and widely in the treatment of various glomerular diseases. More than 60 active ingredients are contained in AS, including AS I–IV, polysaccharides, amino acids and trace elements (Amiri et al., 2020). Chan et al. showed that AS reduces macroalbuminuria in patients with type 2 diabetes, stage 2–3 CKD (Chan et al., 2021). Through this RCT, they successfully evaluated the effects of AS and identified related response predictors for more personalized applications and further researches. Zhai et al. found that AS can ameliorate foot processes effacement and podocyte apoptosis through up-regulation of nephrin, α-dystroglycan, and Bcl-x, which provides an effective treatment for DN (Zhai et al., 2019).

### *Cordyceps sinensis* (BerK.) Sacc. Mycelia (Clavicipitaceae)

As a well-known herb in China and other Asian countries, *Cordyceps sinensis* (BerK.) Sacc. Mycelia (Clavicipitaceae) (CS) is a fungus-caterpillar complex formed after the fungus infects the larva, which plays an important role in TCM in terms of reinforcing kidney and nourishing lung. It is rich in cordycepin, arachidonic acid, cerevisterol, beta-sitosterol, linoleyl acetate, cholesteryl palmitate and other active ingredients (Li Y. et al., 2021). CS has numerous therapeutic effects, including regulating immune disorders, inhibiting cancer cell, inducing cell cycle arrest, apoptosis and autophagy (Chang et al., 2020). Hao et al. demonstrated that CS exhibits a protective effect on podocytes in rats with DN, thereby reducing proteinuria and improving renal function (Hao et al., 2014). Wang et al. reported that CS can significantly inhibit the high expression of P2X7R, and suppress the activation of NLRP3 inflammasome in podocytes, which may be one of the important mechanisms for the therapeutic effects of CS on DN (Wang C. et al., 2018). Additionally, as a bioactive compound of CS, Cordycepin has been proved to be effective in the treatment of various kidney diseases (Han et al., 2020). Hong et al. showed that Cordycepin protects foot processes and cytoskeleton structures of podocytes by suppressing the redistribution of nephrin and inhibiting the activation of p38/JNK signaling pathway (Hong et al., 2015).

### *Salvia przewalskii* Maxim. (Lamiaceae)

*Salvia przewalskii* Maxim. (Lamiaceae) (SP) is a perennial herb plant, which is also called Zidanshen, Ganxishuweicao or Gansudanshen in Chinese. It is mainly distributed in the western of China, including Sichuan, Gansu, Yunnan and Tibet Provinces. According to TCM theory, SP is one of the most important herbs for invigorating blood circulation and eliminating stasis (Yang et al., 2017). Chemical analyses demonstrated that a variety of active ingredients (e.g., tanshinone, isotanshinone, danshenxinkun, danshenol and tanshinlactone) can be extracted from SP. Dai et al. demonstrated the abilities of SP extract from total phenolic acids to reduce the urine protein excretion, preserve the morphology and structure of podocyte, and retain the levels of slit diaphragm proteins on PAN-induced podocyte injury (Dai et al., 2015). Liu et al. reported that the administration of the total phenolic acid extract from SP attenuates OS in PAN-induced rats, ameliorates the increase in the expression of ROS, revises the structure of mitochondria, and finally suppresses podocyte apoptosis (Liu et al., 2016). Ren et al. showed that SP significantly promotes the restoration of typical pattern of F-actin stress fibers in the cell, and down-regulates the protein levels of TLR4, MyD88 and NF-kB (Ren et al., 2018). Furthermore, the protective mechanism of SP on PAN-induced podocyte injury is partially related to the inhibition of TLR4 signaling, which is as strong as tacrolimus in inhibiting TLR4 components expression.

### *Schisandra chinensis* (Turcz.) Baill. (Schisandraceae)

*Schisandra chinensis* (Turcz.) Baill. (Schisandraceae) (SC), also called Wuweizi in Chinese, is a famous magnolia plant that grows in the area of northeastern China for more than two thousand years and the medicinal part is ripe fruit. As an astringent tonic, SC is thought to tonify qi and strengthen yin, as well as promoting the production of body fluid. Nowadays, it is widely used in China, Japan, Korea, and many other east Asian countries to treat neurological diseases and liver diseases (Zhou Y. et al., 2021). Thus far, more than 20 active ingredients have been extracted from SC (e.g., schisandrin B, schisandrol B, anwuligan, schisanhenol and deoxyschizandrin) (Fu et al., 2019). It is reported that the pharmacological effects of SC includes antioxidation, anti-inflammatory, anti-fibrosis, suppression of apoptosis and regulation of neurotransmitters (Zhang M. et al., 2018). Zhang et al. performed *in vitro* and *in vivo* experiments to investigate the effects of SC. They found that SC extract effectively decreases the excretion rate of urine albumin in STZ-induced DN, attenuates glomerulosclerosis, preserves the integrity of slit diaphragm and finally protects against podocyte injury through suppressing EMT (Zhang et al., 2012). They also suggested that SC extract reduces the increased expression of transcription factor snail induced by TGF-1, which indicates a potential and valuable role of SC extract in controlling phenotypic changes of podocytes.

### *Kopsia arborea* Blume (Apocynaceae)

*Kopsia arborea* Blume (Apocynaceae) (KA) is a member of *Apocynaceae* family, which is widely distributed throughout Yunnan Province of China. The leaves, stems, twigs and fruits of KA can be used as medicinal materials and have a long history (Wang et al., 2017). KA is traditionally applied in the treatment of edema, tonsillitis, pharyngitis and rheumatism for its cytotoxic activity, anti-inflammatory activity and antitumor activity (Liu et al., 2020). It is reported that a variety of active ingredients have been extracted from the stems of KA (e.g., kopsioffines A-C, kopsiofficines H-L, erythratinone, erythratine, erysotine, cristanine E) (Xie TZ. et al., 2020). Similar to its stems, the twigs and leaves of KA are also rich in monoterpenoid indole alkaloids (e.g., kopsinidines C-E, 12-methoxychanofruticosinic acid, chanofruticosinic acid, methyl chanofruticosinate, erthrodiol and β-amyrin) (Zeng et al., 2017). Wang et al. evaluated the protective effects of isolated compounds from KA *in vitro* and found that they can antagonize HG-induced podocyte injury activity (Wang et al., 2019).

### *Atractylodes lancea* (Thunb.) DC. (Asteraceae)

*Atractylodes lancea* (Thunb.) DC. (Asteraceae) (AL), a valuable Chinese herbal medicine recorded in Sheng Nong’s Herbal Classic, is widely used in the East Asia as a aromatic, diuretic and stomachic drug for the treatment of kidney-related edema, rheumatic diseases, digestive disorders, and night blindness for thousands years (Zhang WJ. et al., 2021). Chemical analyses demonstrated that abundant pharmacological ingredients have been extracted from AL, and the main active ingredients are a variety of sesquiterpenoids and alkynes, including atractylon, atractyloside A, tractylenolides I, II, III and 8-epiasterolid (Xu et al., 2018). The pharmacological effects of AL include antioxidative, gastroprotective, anti-inflammatory, anti-allergic, antibacterial, antiviral, anticancer and neuroprotective activities (Cheng et al., 2016). Chen et al. showed that AL enhances the expressions of podocin and nephrin, as well as CD2AP and α-Actinin-4, being consistent with its effect on reducing proteinuria (Chen L. et al., 2021). They also suggested that AL can prevent podocyte injury in fructose-induced rats possibly by reducing OS, and those effects are related to the inhibition of TRPC6/p-CaMK4 signaling.

### *Panax notoginseng* (Burkill) F.H.Chen (Araliaceae)

As one of the most popular Chinese herbal medicines in China, the roots of *Panax notoginseng* (Burkill) F.H.Chen (Araliaceae) (PN) has been included in the list of health Chinese herbs with drug and food properties announced by the National Health Commission of China. The best area to plant PN is mainly distributed in Wenshan Zhuang and Miao Autonomy district, which is located in the southeast of Yunnan Province. According to TCM theory, PN is the most important herb to promote blood circulation, stop bleeding, relieve swelling and pain. Besides, it is regarded as the scavenger for blood vessel in modern medicine (Wu et al., 2018). Recently, PN is widely used both in the prevention and treatment of various cardiovascular, cerebrovascular and hematological diseases, such as heart failure, myocardial infarction and cerebral infarction (Fu et al., 2021). Experimental studies have shown that PN has diverse pharmacological activities with notoginsenosides, triterpenoid saponins and dencichine as its main active ingredients (Yao et al., 2019), including anti-inflammatory, anti-obesity, antioxidative, antiplatelet, antihyperglycemic, antihypertensive, and anti-depression properties (Xie et al., 2018b; Zhang C. et al., 2021). Shi et al. proposed that PN attenuates the excretion of proteinuria, improves podocyte injury, and slows the progress of DN both *in vivo* or *in vitro* (Shi et al., 2016). Xie et al. suggested that PN ameliorates proteinuria and podocyte EMT in STZ-induced rats through inhibiting Wnt/β-catenin signaling pathway, which emphasizes a promising novel treatment for DN and other kidney diseases in connection with podocyte injury (Xie L. et al., 2020).

### *Abelmoschus manihot* (L.) Medik. (Malvaceae)

*Abelmoschus manihot* (L.) Medik*.* (Malvaceae) (AM) is an annual herbal flowering plant in the family of *Malvaceae*, which is also named as “Huangkui” in TCM. It is widely distributed in eastern Europe, as well as temperate and subtropical regions of Asia. More than 128 phytochemical constituents have been extracted and identified from the flowers, stems and leaves of AM (e.g., flavonoids, amino acids, polysaccharides, steroids, organic acids and volatile oils) (Luan et al., 2020). Thus far, many studies have demonstrated that AM exerts a plethora of biological properties, including anti-inflammatory, anti-oxidative, anticonvulsant, antiplatelet, neuroprotective, and immunomodulatory effects (Pan et al., 2018). It is worth mentioning that Huangkui capsule, known as the ethanol extract of the flower in AM, has been used to treat patients with proteinuria and other kidney diseases (Chen et al., 2016). Since Tu et al. reported that Huangkui capsule improves renal inflammation and glomerular injury in ADR-induced rats by reducing the infiltration and activation of macrophages *via* inhibiting p38MAPK signaling pathway (Tu et al., 2013), the therapeutic effects of AM on podocyte injury have been reported frequently. Kim et al. found that the therapeutic effects of AM extracts on STZ-induced podocyte injury are closely related to the improvements of autophagy activity and mitochondrial function (Kim et al., 2018). Likewise, Liu et al. provided a better understanding of the total flavones of AM in ameliorating HG-induced podocyte pyroptosis and injury, which is connected with the regulation of NLRP3-inflammasome activation and PTEN/PI3K/AKT signaling (Liu BH. et al., 2021).

## Compound TCM Prescriptions

### Zhenwu Decoction

Zhenwu Decoction (ZWD) is a famous compound prescription created by Zhang Zhongjing in the Han dynasty. ZWD is composed of five herbs, including *Aconitum carmichaelii* Debeaux (Ranunculaceae), *Wolfiporia extensa* (Peck) Ginns (Polyporaceae), *Atractylodes macrocephala* Koidz. (Asteraceae), *Paeonia lactiflora* Pall*.* (Paeoniaceae) and *Zingiber officinale* Roscoe (Zingiberaceae) in Table 3. For thousands of years, it has been widely applied in the treatment of various edema diseases by warming yang to promote diuresis. Modern medical experiments have shown that ZWD has effects of alleviating proteinuria, ameliorating renal function, reducing oxidative lesions and relieving inflammatory damage (Liang et al., 2014). For example, Liu et al. used ZWD in the treatment of podocyte injury. They found that ZWD reveals well therapeutic effects in IgAN-induced podocyte injury both *in vivo* and *in vitro*, and the underlying mechanism might involve the inhibition of PPARγ/NF-κB pathway (Liu B. et al., 2018). They also demonstrated that ZWD can further optimize the therapeutic effects on MN by ameliorating kidney function, improving podocyte injury and inhibiting kidney inflammation. The potential mechanism is closely related to the inhibition of NF-κB pathway and NLRP3 inflammasome (Liu et al., 2019).

**TABLE 3:** Constituents of ZWD.

Number	Chinese herbal medicine	Scientific name	Proportion
1	Fu zi	*Aconitum carmichaelii* Debeaux (Ranunculaceae)	3
2	Fu ling	*Wolfiporia extensa* (Peck) Ginns (Polyporaceae)	3
3	Bai zhu	*Atractylodes macrocephala* Koidz. (Asteraceae)	2
4	Bai shao	*Paeonia lactiflora* Pall. (Paeoniaceae)	3
5	Sheng jiang	*Zingiber officinale* Roscoe (Zingiberaceae)	3

### Huaiqihuang Granules

Huaiqihuang Granules (HQH), a mixture of Chinese herbs, are mainly composed of *Trametes robiniophila* Murr (Polyporaceae), *Lycium chinense* Mill*.* (Solanaceae) and *Polygonatum sibiricum* Redouté (Asparagaceae) in Table 4. HQH acts to tonify qi and nourish yin. It has been widely used in the treatment of primary nephrotic syndrome and other kidney diseases. Some experiments have shown that HQH has renoprotective functions. For example, Zhu et al. investigated the therapeutic role of HQH in podocyte injury caused by ADR. They reported that its therapeutic effect is closely related to the inhibition of inflammatory cytokines (Zhu et al., 2011). Li et al. successfully constructed podocyte damage model using tunicamycin-induced MPC5 cells, and treated them with HQH at different concentrations. They found that the application of HQH can decrease the harmful effects, including promoting cell proliferation, suppressing cell apoptosis, and improving the expressions of podocin, nephrin, and synaptopodin (Li et al., 2016). Liu et al. used HQH in the treatment of ADR-induced nephropathy rats. They showed that it ameliorates renal impairment and podocyte injury by increasing nephrin expression and inhibiting NF-κB signaling pathway, which is connected to the down-regulation of p-NF-κB p65 and p-IκBα (Liu et al., 2017). Besides, Li et al. found that the protective effects of HQH on hyperglycemia-induced podocyte dysfunction are obviously observed. They reported that HQH is able to suppress cell apoptosis, promote mitochondrial dysfunction and ERS in podocytes (Li TX. et al., 2017).

**TABLE 4:** Constituents of HQH.

Number	Chinese herbal medicine	Scientific name	Proportion
1	Huai er jun zhi	*Trametes robiniophila* Murr (Polyporaceae)	4.6
2	Gou qi	*Lycium chinense* Mill. (Solanaceae)	3
3	Huang jing	*Polygonatum sibiricum* Redouté (Asparagaceae)	2.4

### Baoshenfang

Baoshenfang (BSF) is a Chinese herbal formula for the treatment of diabetic podocyte injury. It is created by Zhao wenjing research group of Beijing Hospital of TCM, Capital Medical University. BSF consists of *Astragalus mongholicus* Bunge (Fabaceae), *Ligustrum lucidum* W.T.Aiton (Oleaceae), *Salvia miltiorrhiza* Bunge (Lamiaceae), leeches, and scorpions in specific proportions. Cui et al. found that BSF has therapeutic effects in decreasing 24 h urinary protein, SCr and BUN levels in DN rats, and the underlying mechanism is closely related to inhibition of the NOX-4/ROS/p38 pathway (Cui et al., 2019a). Additionally, subsequent continuity research showed that BSF can significantly attenuate OS and apoptosis in podocytes, increase cell viability, and inhibit actin cytoskeleton rearrangement. More importantly, the effect of BSF on podocyte apoptosis is partly through activating the PI3K/AKT pathway (Cui et al., 2019b).

### Tongxinluo

Tongxinluo (TXL) is a new kind of Chinese herbal compound that serves to tonify qi, promote blood circulation, activate meridians and relieve pain. It includes a group of valuable Chinese herbal medicines, such as *Panax ginseng* C.A.Mey. (Araliaceae), *Paeonia lactiflora* Pall. (Paeoniaceae), borneol, centipede and frankincense*.* TXL has exhibited its powerful antioxidant effect in the treatment of cardiovascular diseases and cerebrovascular diseases in China (Ma et al., 2019). Recently, more and more studies have proved that TXL can significantly decrease proteinuria and ameliorate renal dysfunction. Luo et al. found that TXL protects against hypertension-induced podocyte injury by exerting antioxidative, antifibrotic and anti-inflammatory activities to inhibit OS and the activation of FoxO1 signaling (Luo et al., 2016). Cui et al. showed that TXL promotes the expression of nephrin in diabetic rats and HG cultured podocytes by inhibiting the activation of notch1/snail pathway, which may be an important mechanism in treating DN (Cui et al., 2015). Subsequent continuity research demonstrated that TXL also attenuates OS after podocyte injury in DN. This regulation is closely related to its antioxidant effect and the inhibition of P38 and caspase-3 (Cui et al., 2016).

## Acupuncture and Moxibustion

### Acupuncture

As an integral part of complementary and alternative medicine, acupuncture has been used for thousands of years in China and gradually been accepted as an important part of therapy in Western countries (Jin et al., 2020). Accumulating evidences indicate that acupuncture is safe and has great clinical efficacy in the treatment of various diseases, including gastrointestinal diseases, neurodegenerative diseases, cardiovascular diseases, chronic pain, insomnia and CKD (Song et al., 2019). Scientific researches into the mechanisms of acupuncture have been rapidly growing in the past several decades, which prove that acupuncture can exert anti-inflammatory, analgesic, antiapoptotic, antioxidative, neuroprotective and antidepressant effects (Huang and Hsieh, 2021). Zhang et al. demonstrated that “Tiaolipiwei” acupuncture, including Zhongwan (CV12), Quchi (LI11), Hegu (LI4), Zusanli (ST36), Yinlingquan (SP9), Xuehai (SP10), Diji (SP8), Sanyinjiao (SP6), Fenglong (S40) and Taichong (LR3), significantly reduces proteinuria, improves the density of slit diaphragms, and ameliorates podocyte injury in DN rats *via* promoting the expressions of nephrin, CD2AP, and podocalyxin (Zhang Z. et al., 2018). An et al. successfully performed “Qufeng Tongluo” acupuncture on Fengmen (BL-12) and Shenshu (BL-23) for the treatment of cBSA-induced glomerulonephritis. Subsequently, they found that “Qufeng Tongluo” acupuncture restores renal function, improves podocyte injury, and reduces renal sympathetic nerve activity through inhibiting Erk1/2 MAPK pathway (An et al., 2014).

### Moxibustion

As an important part of TCM, moxibustion is the process of burning the herb moxa (Schlaeger et al., 2018). It is widely accepted around the world for those advantages, such as no pains, no injuries, fewer side effects, easy to operate and moderate cost. Compared to acupuncture, moxibustion is motivated by warm stimulation, which involves using the heat of burning moxa to stimulate acupoints. When it comes to moxibustion, moxa can be rolled into stick form placed directly on the skin at a distance, or placed on an acupuncture needle to warm acupuncture points (Huang et al., 2017). For thousands of years, moxibustion has been used for various diseases, including dysmenorrhea, inflammatory bowel disease, skin diseases, and nervous system diseases (Stein, 2017). Experimental results show that the mechanisms of moxibustion mainly contain the thermal effects, radiation effects, and pharmacological actions by activating local specific receptors and heat sensitive immune cells (Deng and Shen, 2013). Li et al. evaluated the potential therapeutic effects of mild Shenshu moxibustion (BL-23) and Geshu moxibustion (BL-17) in FSGS rats. They found that moxibustion at Shenshu and Geshu acupoints can decrease urinary protein levels and improve renal function through alleviating podocyte injury and inhibiting renal interstitial fibrosis (Li Y. et al., 2017).

## Conclusion and Perspectives

Proteinuria is considered as a major healthcare problem affecting several hundred million people worldwide. Podocytes are known to be terminally divided cells that cover the outer layer of GBM. As a major contributor to proteinuria, podocyte injury underlies a variety of glomerular diseases and becomes the challenge to patients and their families in general. Although serious social harm is caused by this disorder, there are no effective treatments for patients with podocyte injury. Besides, the mechanisms of podocyte injury are multiple and complex. Therefore, it is difficult to prove which mechanism plays a decisive role in the occurrence and development of podocyte injury. Currently, the broader goal suggests that treatment for podocyte injury involves more than simple reduction in the amount of protein released from urine. Thus, joint efforts by many fields are needed to achieve a satisfactory result.

Obviously, TCM shows advantages in the treatment of podocyte injury. In clinical practice, TCM is frequently used alone or in conjunction with western pharmacotherapy for treatment of kidney diseases, especially in China and other Asian countries. Even in the West, many patients seek out alternative therapies such as herbal medicines, acupuncture and moxibustion. Recently, researches regarding active ingredients extracted from Chinese herbs are the most rapidly growing area among these various fields. Although herbs and compound prescriptions have extraordinarily complex compositions, the multi-target effects of TCM can compensate for the inevitable limitations. Furthermore, TCM formulas involve multiple signal pathways to regulate the expressions of a series of related proteins, and finally achieve a synergistic therapeutic effect, which cannot be accomplished by a single chemical drug. Accordingly, TCM for the treatment of podocyte injury has great prospects for further development.

In this review, we summarized the protective effects of TCM on podocyte injury, including active ingredients, herbs, compound prescriptions, acupuncture and moxibustion. With respect to active ingredients, AS-IV, TP, BBR, and Rg1 are highlighted. In herbs, *Astragalus mongholicus* Bunge (Fabaceae), *Cordyceps sinensis* (BerK.) Sacc. Mycelia (Clavicipitaceae), *Salvia przewalskii* Maxim. (Lamiaceae), and A*belmoschus manihot* (L.) Medik. (Malvaceae) are commonly used and studied medicines. When it comes to compound prescriptions, the herbal medicines are mainly composed of blood-activating medicines, followed by kidney and spleen tonifying medicines. Although reports on acupuncture and moxibustion in the treatment of podocyte injury are few, it still can be a promising research direction. As for research models, HG-induced podocyte injury, STZ-induced podocyte injury, and ADR-induced podocyte injury are the most frequently used models. Regarding mechanisms, AMPK, PPARγ, and NF-κB, as well as SIRT1/PGC-1α pathway are research hotspots. These findings can not only guide and inspire researchers to explore new and effective treatments for podocyte injury, but also make great contributions to the modernization of TCM. While efforts still need to be deployed to give full play to the advantages of TCM for podocyte injury. Accordingly, we advise the following actions:

1) Podocyte injury requires long-term medication. Although the safety and stability have been tested, the potential cumulative toxicity caused by TCM in metabolic and excretory organs should be considered, including hepatotoxicity and nephrotoxicity.
2) In spite some of herbs indeed have protective effects on podocyte injury, only simply estimated changes of relevant proteins are observed. We still have very limited knowledge about the active ingredients and clear mechanisms of TCM, which should be studied thoroughly *via* cellular and molecular technology.
3) Researches regarding TCM continue to be widely published in journals with low influence and lack of high quality clinical trials. Thus, this research field requires additional in-depth studies to promote better development.
4) Single *in vitro* cell models or *in vivo* models reported in the articles cannot accurately simulate the complex physiological and pathological mechanisms of podocyte injury. So, well-designed RCTs are expected to be performed to confirm the efficacy and safety before TCM can be used as therapeutic alternatives for patients.
5) Scientific clarification and quality control of chemical compositions of Chinese herbs and compound prescriptions that can be applied in the treatment of podocyte injury are worrying. Therefore, We should establish uniform drug quality control standards and identify optimal drug dose through analytical methods such as HPLC and other chromatography.
6) Non-drug therapy should get more attention. For example, appropriate therapies combined with acupuncture and various traditional sports may improve the efficacy and avoid the accumulation poisoning, which need further researches.

In conclusion, further efforts are required to improve our understanding of the mechanisms of TCM. Just like the great discovery of artemisinin extracted from the Chinese herbal medicine *Artemisia annua* L. (Asteraceae), there is still a long way to go to find satisfactory drugs from TCM. We believe that the road is rough, while the future is bright.

## Glossary

GFB: Glomerular filtration barrier
CKD: Chronic kidney disease
ESRD: End-stage renal disease
TCM: Traditional Chinese medicine
MCD: Minimal change disease
FSGS: Focal segmental glomerulosclerosis
MN: Membranous nephropathy
DN: Diabetic nephropathy
LN: Lupus nephritis
GBM: Glomerular basement membrane
EMT: Epithelial-mesenchymal transition
AS-IV: Astragaloside IV
HG: High glucose
STZ: Streptozotocin
TRPC6: Transient receptor potential cation channel 6
FoxO1: Forkhead transcription factor O1
PPARγ: Peroxisome proliferator-activated receptor γ
PAN: Puromycin aminonucleoside
TP: Triptolide
ADR: Adriamycin
TGF-β1: Transforming growth factor beta 1
NF-κB: Nuclear factor kappa-B
p-IκBα: Phospho-inhibitor of NF-κB
BBR: Berberine
ERS: Endoplasmic reticulum stress
OS: Oxidative stress
ROS: Reactive oxygen species
PI3K: Phosphatidylinositol 3-kinase
AKT: Protein kinase B
4EBP1: 4E-binding protein 1
mTOR: Mammalian target of rapamycin
P70S6K: P70 ribosomal S6 kinase
CUR: Curcumin
Ang II: Angiotensin II
Bcl2: B-cell lymphoma-2
UVRAG: UV radiation resistance associated gene
Drp1: Dynamin-related protein 1
Nrf2: Nuclear factor-E2-related factor
AMPK: Adenosine monophosphate-activated protein kinase
MAPK: Mitogen-activated protein kinase
EMO: Emodin
MGO: Methylglyoxal
CAT: Catalpol
SOD: Superoxide dismutase
MDA: Malondialdehyde
LDH: Lactate dehydrogenase
SIRT1: Sirtuin
MRP2: Multidrug resistance-associated protein 2
NADPH: Nicotinamide adenine dinucleotide phosphat
LUT: luteolin
NLRP3: NOD-like receptor family and pyrin domain-containing protein 3
HPS: Hyperoside
PGC-1α: Peroxlsome proliferator-activated receptor-γ coactlvator-1α
OPA1: Optic atrophy
AKI: Acute kidney injury
IR: Ischemia/reperfusion
SAL: Salidroside
PF: Paeoniflorin
ANGPTL4: Angiopoietin-like Protein4
RES: Resveratrol
HFD: High-fat diet
NOD: Non obesity diabetes
BG: Blood glucose
BUN: Blood urea nitrogen
SCr: Serum creatinine
hUCMSCs: Human umbilical cord mesenchymal stem cells
MCP-1: Monocyte chemoattractant factor-1
Rg1: Ginsenoside Rg1
BA: Baicalin
AS: Astragalus
RCT: Randomised controlled trial
CS: Cordyceps sinensis
P2X7R: Purinergic 2X7 receptor
JNK: C-Jun N-terminal kinase
SP: Salvia przewalskii
TLR4: Toll-like receptor 4
SC: Schisandra chinensis
KO: Kopsia officinalis
AR: Atractylodis Rhizoma
CaMK4: Calcium/calmodulin-dependent protein kinase IV
PN: Panax notoginseng
AM: Abelmoschus manihot
PTEN: Phosphate and tensionhomology
ZWD: Zhenwu Decoction
HQH: Huaiqihuang Granules
IκBα: Inhibitor kappa B alpha
BSF: Baoshenfang
TXL: Tongxinluo
FoxO1: Forkhead box O1
CD2AP: CD2-associated protein
Erk1: Extracellular regulated protein kinases 1
